# Prevention of Cholangitis by Spontaneously Dislodging Biliary Stent After an Endoscopic Procedure in Patients With Asymptomatic Bile Duct Stones

**DOI:** 10.1002/deo2.70194

**Published:** 2025-08-23

**Authors:** Shinichi Nihei, Sho Hasegawa, Yu Honda, Yuma Yamazaki, Takeshi Iizuka, Yusuke Kurita, Kunihiro Hosono, Masato Yoneda, Kensuke Kubota, Atsushi Nakajima

**Affiliations:** ^1^ Department of Gastroenterology and Hepatology Yokohama City University Kanagawa Japan

**Keywords:** bile ducts, cholangitis, endoscopic retrograde cholangiopancreatography, gallstones, stents

## Abstract

**Objective:**

To evaluate the effectiveness of spontaneously dislodging biliary stent (SDBS) placement in preventing acute cholangitis after endoscopic retrograde cholangiopancreatography (ERCP) in patients with asymptomatic bile duct stones.

**Methods:**

This retrospective, single‐center study included 63 patients (mean age, 73 ± 11 years; 43 men) who underwent ERCP for asymptomatic bile duct stones at our institute between April 2022 and May 2024; they were categorized into the SDBS (33 patients) and non‐stent (30 patients) groups. Stone removal was performed in all cases, and complete stone clearance was achieved. Post‐procedure cholangitis was diagnosed based on the Tokyo Guidelines 2018. The primary endpoint was post‐procedural acute cholangitis on day one. Secondary endpoints included cholangitis‐related factors, type of device used, procedure time, and adverse events other than cholangitis. Multivariate analysis was performed to identify factors associated with post‐procedure cholangitis.

**Results:**

No significant differences were observed in prior cholecystectomy, bile duct diameter, or the number of stones between the two groups. The overall incidence of post‐procedure cholangitis was 17.4%. The incidence was significantly lower in the SDBS group than in the non‐stent group (6% vs. 30%; *p =* 0.01). No significant differences were observed in procedure time, incidence of complications such as post‐ERCP pancreatitis, or postoperative hospital stay between the SDBS and non‐stent groups. Multivariate analysis identified the absence of an SDBS (odds ratio, 6.09; 95% confidence interval, 1.04–35.6, *p =* 0.04) as an independent factor associated with post‐procedure cholangitis.

**Conclusion:**

SDBS placement may be effective in preventing cholangitis after ERCP in asymptomatic patients with bile duct stones.

## 1 Introduction

The diagnosis of asymptomatic bile duct stones is becoming more common owing to advancements in imaging technology [1]. Given the risks of pancreatitis and cholangitis, various guidelines recommend the removal of asymptomatic bile duct stones via endoscopic retrograde cholangiopancreatography (ERCP) [2, 3, 4]. However, the incidences of ERCP‐related complications are reportedly higher for asymptomatic stones than symptomatic ones [5, 6]. Common ERCP‐related complications include post‐ERCP pancreatitis, perforation, bleeding, and cholecystitis. Moreover, post‐ERCP cholangitis occasionally poses a challenge, with an incidence rate of 0.5%–3% [7, 8, 9]. Clinical symptoms of post‐ERCP cholangitis include fever, abdominal pain, and jaundice; severe cases can sometimes be fatal [10], which underscores the importance of prevention. Known preventive measures for post‐ERCP cholangitis include prophylactic antibiotic administration [10] and CO_2_ cholangiography [10, 11]; however, the efficacy of these modalities has not been firmly established.

Recently, spontaneously dislodging biliary stents (SDBSs) have been developed as a preventive measure against post‐ERCP cholangitis. However, studies evaluating the utility of SDBSs for bile duct stones are limited [12]. We hypothesized that SDBSs could be effective in preventing duodenal papillary edema after bile duct stone removal. Accordingly, we investigated the utility of SDBS placement in preventing acute cholangitis after ERCP in patients with asymptomatic bile duct stones.

## 2 Methods

### 2.1 Study Population

**FIGURE 1:**
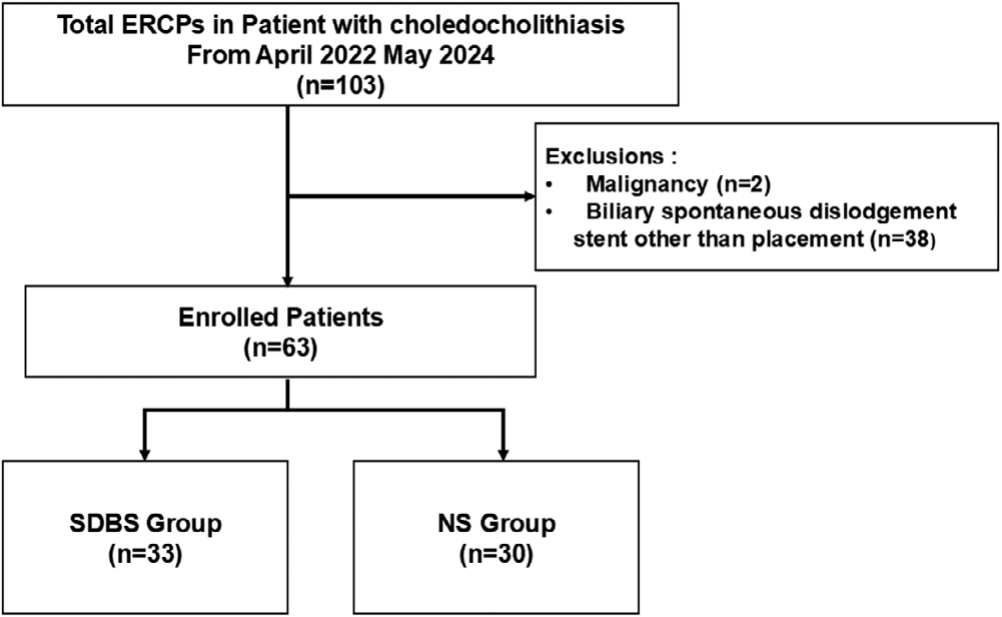
Flowchart of patient selection. SDBS, spontaneously dislodging biliary stent; NS, non‐stent.

Between April 2022 and May 2024, 103 ERCP procedures were performed for asymptomatic bile duct stones at the Yokohama City University Hospital. Among them, patients with malignant tumors or those treated with nonspontaneous bile duct stents were excluded; the remaining 63 patients were included in our study. The patients were divided into SDBS (33 patients) and non‐stent (30 patients) groups (Figure 1). Stone removal was performed in all cases, and complete stone clearance was achieved.

This study was approved by the Institutional Review Board of Yokohama City University Hospital (F240100033) and was conducted following the latest version of the Declaration of Helsinki and the ethical standards established by the Yokohama City University Clinical Research Review Committee. This retrospective study utilized only medical data and did not infringe upon participant privacy. Consent was obtained from all participants using an opt‐out method. Patients who declined to participate were excluded from the study.

### 2.2 ERCP Procedure

ERCP was performed by experienced endoscopists who conducted over 200 procedures annually. Conscious sedation was induced via intravenous midazolam, pentazocine, and diazepam, with propofol administered as needed. All patients were administered intravenous cefmetazole sodium before and 12 h after ERCP. A side‐viewing duodenoscope (TJF‐290 V; Olympus Medical Systems) was used for the procedure.

Following selective bile duct cannulation, 60% amidotriazoic acid was injected to evaluate bile duct stone formation. For untreated papillae, the endoscopists performed endoscopic sphincterotomy (EST), endoscopic papillary balloon dilation (EPBD), or endoscopic papillary large balloon dilation (EPLBD), as appropriate. Additional interventions were performed as required in patients with a history of papillary treatment. For patients with difficult biliary cannulation, pancreatic duct guidewire placement or precut techniques were employed.

**FIGURE 2:**
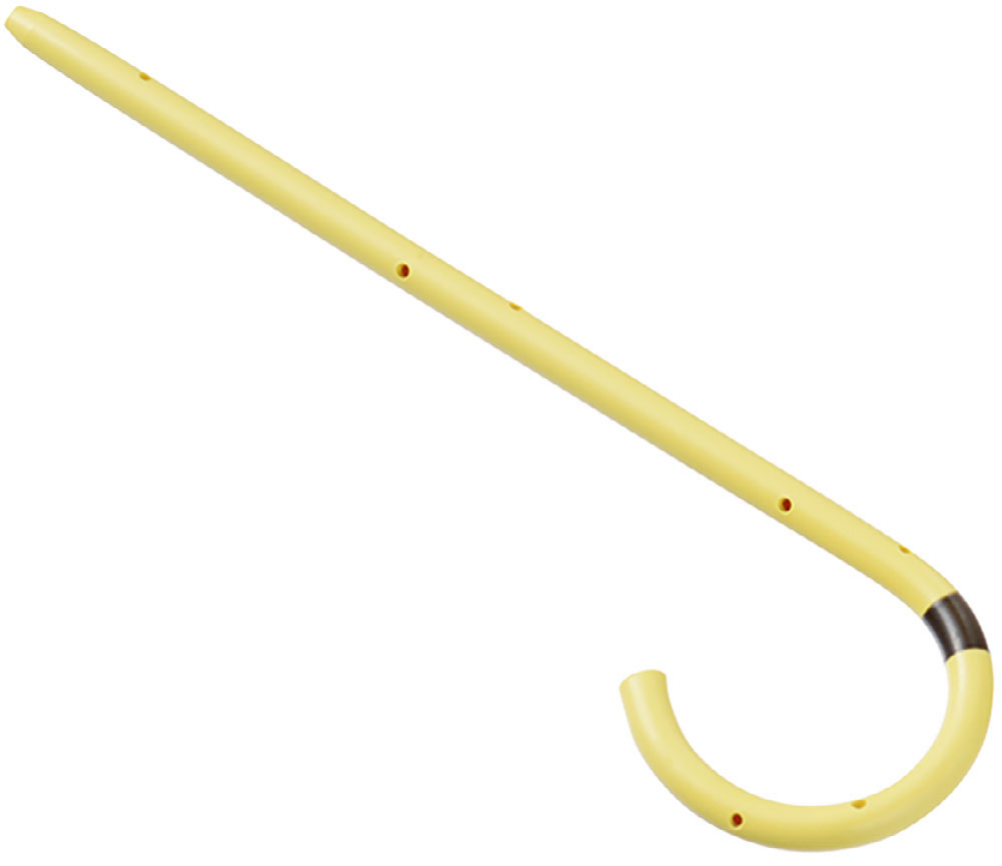
Spontaneously dislodging biliary stent (SDBS). The stent used was a 6‐Fr, 4‐cm polyethylene Pit‐stent (GADELIUS, Tokyo, Japan), featuring a single pigtail and no flange on the bile duct side.

**FIGURE 3:**
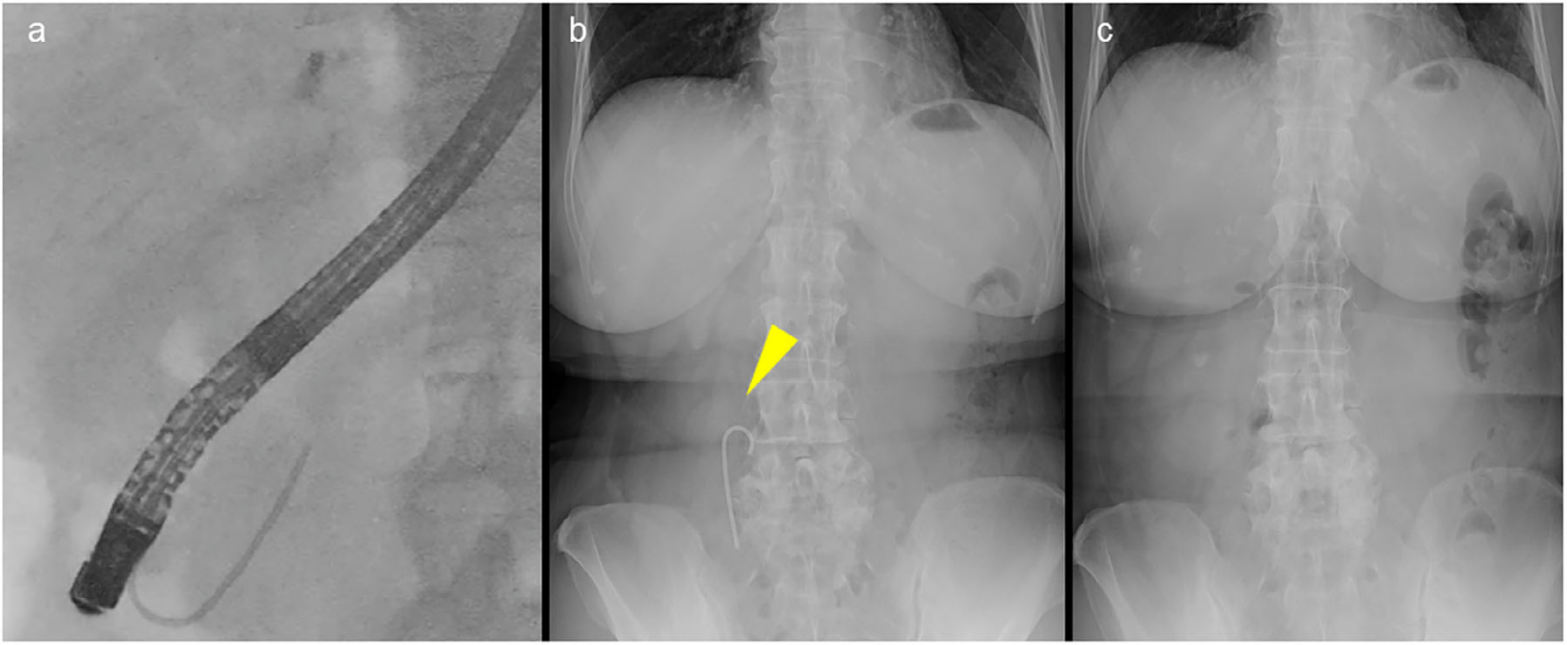
(a) The placement of the spontaneously dislodging biliary stent (SDBS). (b) An X‐ray image captured the day after endoscopic retrograde cholangiopancreatography (ERCP) showing that the SDBS had deviated. (c) X‐ray image captured 3 days after ERCP showing that the stent has disappeared.

Stone removal methods included the use of baskets, balloon catheters, mechanical lithotripters, and electrohydraulic lithotripsy (EHL). After stone extraction, balloon cholangiography was performed to confirm the absence of residual stones. SDBS was placed at the discretion of the endoscopist. Polyethylene stents (6‐Fr diameter, 4 cm length) with a single pigtail (Pit stent; GADELIUS, Tokyo, Japan) were used for bile duct stenting (Figure 2). If the pancreatic duct guidewire method was used, an SDBS was placed at the discretion of the endoscopist. Stent dislodgement was confirmed via abdominal radiography performed on postoperative days 1 and 3 (Figure 3).

### 2.3 Definitions

Asymptomatic bile duct stones were defined as stones that were incidentally detected in patients without symptoms such as abdominal pain or abnormalities in blood tests [13]. Post‐ERCP cholangitis was defined based on the diagnostic criteria for acute cholangitis outlined in the Tokyo Guidelines 2018 [13]. The severity of post‐ERCP cholangitis was assessed based on the severity grading criteria for acute cholangitis outlined in the Tokyo Guidelines 2018 [13]. Briefly, cholangitis was diagnosed when two or more of the following characteristics were observed: fever, evidence of inflammation or jaundice, abnormal liver function tests, or imaging findings indicating bile duct lesions.

An endoscopist who had performed <200 independent ERCP procedures was considered an ERCP trainee [14].

### 2.4 Primary and Secondary Endpoints

Herein, the primary endpoint was the incidence of acute cholangitis on the day after ERCP. Secondary endpoints included factors related to cholangitis (age, median bile duct diameter, and median maximum diameter of bile duct stones), type of device used, procedure time, and adverse events other than cholangitis.

### 2.5 Statistical Analysis

Statistical analyses were performed using JMP Pro 17 software (SAS Institute, Cary, NC, USA). Numerical variables were expressed as mean or median values and compared using Student's t‐tests. Categorical variables were analyzed using the Chi‐squared test or Fisher's exact test. Predictors of post‐ERCP cholangitis were analyzed using logistic regression, and odds ratios and 95% confidence intervals were calculated. All tests were two‐tailed, and *p*‐values <0.05 were considered statistically significant.

## 3 Results

### 3.1 Patient Characteristics

The characteristics of patients of the two groups are summarized in Table 1. The mean age of the cohort was 73 ± 11 years, with a male‐to‐female ratio of 43:20. The diagnoses included 62 cases of common bile duct stones and 1 case of intrahepatic stones. No significant differences were observed in prior cholecystectomy, bile duct diameter (>10 mm), or the number of stones (>2) between the two groups.

**TABLE 1:** Clinical characteristics of patients.

Clinical characteristics of patients	SDBS Group *n* = 33	NS Group *n* = 30	*p*‐Value
Age, mean ± SD, years	72 ± 12	75 ± 10	0.42
Sex, male	22 (66.7)	21 (70)	0.77
Diagnosis			
choledocholithiasis	32 (96.7)	30 (100)	0.25
intrahepatic gallstone	1 (3.0)	0 (0)	0.52
Post cholecystectomy	4 (12.1)	5 (16.7)	0.6
Surgically altered anatomy (%)			
B‐I	0 (0)	1 (3.3)	0.22
B‐II	0 (0)	2 (6.7)	0.08
R‐Y	1 (3)	3 (10)	0.24
CBD diam (>10 mm) (%)	4 (12.1)	6 (20)	0.39
Stones number >2	9 (27.2)	4 (13.1)	0.16
Untreated papilla (%)	21 (63.6)	20 (66.7)	0.8

Abbreviations: CBD, Common bile duct; NS, non‐stent; SD, standard deviation; SDBS, spontaneously dislodging biliary stent.

### 3.2 Clinical Outcomes

As shown in Table 2, there were no significant differences in papillary procedures, including EST, pre‐cut methods, EPBD, or EPLBD, between the two groups. The pancreatic duct guidewire method was used in 27.2% (9/33) of patients in the SDBS group and 26.6% (8/30) of patients in the non‐stent group (*p =* 0.95). Mechanical lithotripsy was performed in 21.2% (7/33) of patients in the SDBS group and 30.0% (9/30) of patients in the non‐stent group (*p =* 0.25). Positive bile cultures were observed in 24.2% (8/33) of patients in the SDBS group and 36.6% (11/30) of patients in the non‐stent group (*p =* 0.28). Trainee operators were involved in 81.8% (27/33) of cases in the SDBS group and 70.0% (21/30) of cases in the non‐stent group (*p =* 0.27). The mean procedure time was 26 ± 12 min in the SDBS group and 29 ± 17 min in the non‐stent group (*p =* 0.36). The average length of postoperative hospital stay was 3 ± 1 days in the SDBS group and 4 ± 1 days in the non‐stent group (*p =* 0.10).

**TABLE 2:** Details of endoscopic retrograde cholangiopancreatography (ERCP) procedures and clinical outcomes.

Clinical outcomes	SDBS Group *n* = 33	NS Group *n* = 30	*p*‐Value
Papillary intervention			
EST	19 (57.6)	15 (50)	0.54
Pre cut	1 (3.0)	1 (3.3)	0.94
EPBD	1 (3.0)	1 (3.3)	0.94
EPLBD	5 (15.1)	7 (23.3)	0.4
Pancreatic duct guidewire method	9 (27.2)	8 (26.6)	0.95
ERCP modalities for stone removal			
Balloon catheter	27 (81.8)	20 (66.7)	0.16
Basket catheter	12 (36.3)	13 (43.3)	0.57
Mechanical lithotripsy	7 (21.2)	9 (30)	0.11
EHL	1 (3.0)	0 (0)	0.25
Bile culture (%)	8 (24.2)	11 (36.6)	0.28
Trainee (%)	27 (81.8)	21 (70)	0.27
Procedure time, mean ± SD, min	26 ± 12	29 ± 17	0.36
Postoperative length of stay, mean ± SD, days	3 ± 1	4 ± 1	0.1

Abbreviations: EHL, Electronic hydraulic lithotripsy; EPBD, Endoscopic papillary balloon dilation; EPLBD, Endoscopic papillary large balloon dilation; ERCP, Endoscopic retrograde cholangiopancreatography; EST, Endoscopic sphincterotomy; NS, non‐stent; SD, standard deviation; SDBS, spontaneously dislodging biliary stent.

### 3.3 Post‐ERCP Complications

The incidences and severity of post‐ERCP complications are summarized in Table 3. The overall incidence rate of post‐ERCP cholangitis was 17.4% (11/63). The incidence of cholangitis was significantly lower in the SDBS group (6.0%, 2/33) than in the non‐stent group (30%, 9/30) (*p =* 0.01). Regarding the severity of cholangitis, all cases in the SDBS group were mild, whereas the non‐stent group included seven mild and two moderate cases. The overall incidence of post‐ERCP pancreatitis was 11.1% (7/63): 6% (2/33) in the SDBS group and 16.6% (5/30) in the non‐stent group (*p =* 0.17). All cases of pancreatitis were mild. Bleeding occurred in one patient in the SDBS group. Perforation or cholecystitis was not observed in either group.

**TABLE 3:** Endoscopic retrograde cholangiopancreatography (ERCP)‐related adverse events.

Complications	SDBS Group *n* = 33	NS Group *n* = 30	*p*‐Value
Post‐ERCP Cholangitis	2 (6.0)	9 (30)	0.01
Mild	2 (6.0)	7 (23.3)	0.04
Moderate	0 (0)	2 (6.67)	0.08
Severe	0 (0)	0 (0)	
Post‐ERCP Pancreatitis	2 (6.0)	5 (16.6)	0.17
Mild	2 (6.0)	5 (16.6)	0.17
Moderate	0	0	
Severe	0	0	
Post‐procedure Bleeding	1 (3.0)	0	0.25
Cholecystitis	0	0	
Perforation	0	0	

Abbreviations: ERCP, endoscopic retrograde cholangiopancreatography; NS, non‐stent; SDBS, spontaneously dislodging biliary stent.

### 3.4 Factors Associated With Post‐ERCP Cholangitis

The results of the univariate and multivariate analyses of factors associated with post‐ERCP cholangitis are presented in Table 4. In the univariate analysis, the use of SDBS was the only independent factor that significantly reduced the incidence of post‐ERCP cholangitis (*p =* 0.02). Other factors, including advanced age (>75 years), prior cholecystectomy, involvement of trainee operators, procedure time (>30 min), positive bile culture, bile duct diameter (>10 mm), the number of stones (>2), untreated papilla, EST, EPLBD, ballon catheter use, basket catheter use, and mechanical lithotripsy were not identified as contributors to post‐ERCP cholangitis. In the multivariate analysis, SDBS placement (odds ratio 0.18, 95% confidence interval 0.02–0.94, *p =* 0.04) was the sole independent factor associated with post‐ERCP cholangitis.

**TABLE 4:** Results of the univariate and multivariate analyses of risk factors.

		Univariate analysis			Multivariate analysis	
	Odds ratio	95% CI	*p*‐Value	Odds ratio	95% CI	*p*‐Value
Age >75 years	3.0	0.33–26.7	0.27	3.03	0.37–64.9	0.31
Prior cholecystectomy	2.25	0.24–20.4	0.43			
Trainee	0.56	0.13–2.40	0.44			
SDBS	0.18	0.03–0.98	0.02	0.18	0.02–0.94	0.04
Procedure time >30min	1.23	0.29–5.13	0.76			
Bile culture	1.47	0.31–6.90	0.62			
CBD diameter (>10 mm)	1.10	0.27–4.55	0.88			
Number of stones >2	1.71	0.36–8.14	0.50			
Untreated papilla	5.40	0.62–46.9	0.07	6.19	0.44–175.9	0.17
EST	1.90	0.43–8.46	0.38	1.50	0.12–27.6	0.75
EPLBD	1.37	0.12–14.77	0.79			
Balloon catheter	1.92	0.35–10.38	0.42			
Basket catheter	1.22	0.30–4.89	0.77			
Mechanical lithotripsy	2.02	0.41–9.80	0.39	2.79	0.27–36.0	0.37

Abbreviations: CBD, Common bile duct; CI, confidence interval; EPLBD, Endoscopic papillary large balloon dilation; EST, Endoscopic sphincterotomy; SDBS, spontaneously dislodging biliary stent.

## 4 Discussion

Herein, we investigated the utility of SDBS placement in preventing post‐ERCP cholangitis in patients with asymptomatic bile duct stones. The SDBS group showed a significantly lower incidence of post‐ERCP cholangitis than the other group. Furthermore, univariate and multivariate analyses identified SDBS placement as the sole independent factor associated with post‐ERCP cholangitis. SDBS may be helpful in preventing acute cholangitis after ERCP in patients with asymptomatic bile duct stones. Therefore, SDBS should be considered when an endoscopic procedure with asymptomatic biliary stones is planned.

Post‐ERCP cholangitis can be fatal; however, no standardized preventive measures have been established. Previous reports recommended prophylactic antibiotic administration before ERCP in patients with suspected biliary obstruction, such as bile duct stones or a history of liver transplantation [10, 15]. All patients received prophylactic antibiotics before ERCP; however, 17.4% (11/63) of patients developed post‐ERCP cholangitis. The reported incidence of post‐ERCP cholangitis ranges from 0.5% to 3%, which is lower than that observed in our study. This discrepancy may be attributed to the use of the Tokyo Guidelines 2018 for the diagnosis and the inclusion of only patients with asymptomatic bile duct stones in this study. Currently, diagnostic criteria and severity grading for post‐ERCP cholangitis are described only in the European Society of Gastrointestinal Endoscopy (ESGE) guidelines. According to the ESGE guidelines, Cotton et al. defined post‐ERCP cholangitis as fever ≥38°C persisting for more than 24 h, accompanied by biliary stasis [16]. Additionally, severity grading was based on the Tokyo Guidelines 2018 to ensure consistency after considering the variability in the diagnosis and severity assessment of post‐ERCP cholangitis [17].

Asymptomatic bile duct stones are known to be associated with a higher incidence of ERCP‐related complications compared with symptomatic stones [5, 6]. Our study only included asymptomatic patients with bile duct stones, which may have contributed to the higher incidence of post‐ERCP cholangitis.

ERCP for bile duct stones is associated with multiple risk factors of post‐ERCP cholangitis. Procedures such as EST, EPBD, and stone removal are known to increase the incidence of post‐ERCP cholangitis due to duodenal papillary edema and the reflux of intestinal fluids [18, 19, 20]. In the present study, the incidence of ERCP‐related cholangitis was significantly reduced in patients who underwent SDBS placement after stone removal. The efficacy of bile duct stents after mechanical lithotripsy for stones less than 10 mm has been reported [12], which is consistent with our findings. SDBS may prevent duodenal papillary edema. Furthermore, our findings suggest that spontaneous bile duct stent placement may be effective in preventing post‐ERCP cholangitis, regardless of the stone size or device used. When limited to the 41 patients with untreated papillae, the incidence of post‐ERCP cholangitis was significantly lower in the SDBS group (9.5%, 2/21) compared to the non‐stent group (40.0%, 8/20) (*p =* 0.01). These findings indicate that the placement of an SDBS may be beneficial in preventing post‐ERCP cholangitis in patients with untreated papillae.

SDBSs have the advantage of not requiring additional endoscopic procedures for stent removal compared with conventional non‐spontaneous bile duct stents. On postoperative day 1, the SDBSs remained in place in 66.7% (22/33) of cases, and by day 3, this dropped to 3.0% (1/33). These cases were associated with periampullary diverticula in which the pigtail of the stent was caught, which highlights the need for caution in such cases. However, in most cases, spontaneous dislodging occurred the following day, suggesting high safety.

The optimal treatment strategy for asymptomatic bile duct stones remains controversial. The ESGE and National Institute for Health and Care Excellence (NICE) guidelines recommend ERCP for asymptomatic bile duct stones because of the risk of cholangitis [2, 3, 4]. However, ERCP for asymptomatic bile duct stones reportedly leads to a higher incidence of post‐ERCP pancreatitis than that for symptomatic stones [21]. In our study, post‐ERCP pancreatitis occurred in 11.1% (7/63) of cases, which was similar to previous reports. Since asymptomatic bile duct stones are not always associated with bile stasis, a narrow papillary opening potentially makes cannulation difficult, increasing the risk of post‐ERCP pancreatitis. Additionally, 76.1% (48/63) of the procedures were performed by trainees in our study, which might have contributed to the higher incidence of post‐ERCP pancreatitis. Recent studies have reported that when performed by experienced endoscopists, the safety of ERCP for asymptomatic bile duct stones is comparable with that for symptomatic stones [22]. Thus, ERCP for asymptomatic bile duct stones should be performed by experienced endoscopists to mitigate the risk of post‐ERCP pancreatitis.

This study has some limitations. First, this was a single‐center retrospective study. Further prospective studies with larger sample sizes are warranted. Second, the use of the Tokyo Guidelines 2018 for diagnosing post‐ERCP cholangitis may have influenced the results. The variability in the diagnostic criteria necessitates further studies to establish consistent clinical definitions and diagnostic criteria for post‐ERCP cholangitis. Third, the study population was heterogeneous, as it was not limited to patients with untreated papillae. In the future, a prospective study focusing exclusively on patients with untreated papillae is warranted.

In conclusion, as SDBS placement is a useful strategy to prevent post‐ERCP cholangitis in patients with asymptomatic bile duct stones, we should consider it a preventive measure.

## Conflicts of Interest

The authors declare no conflicts of interest.

## Ethics Statement

The study was conducted in accordance with the Declaration of Helsinki and approved by the Institutional Review Board of Yokohama City University Hospital (F240100033). In this retrospective study, only medical data were used, and the privacy of the participants was upheld.

## Consent

Consent was obtained from all participants using an opt‐out method. Patients who declined to participate were excluded from the study.

## Clinical Trial Registration

N/A.
